# Extent With Modification: Leg Patterning in the Beetle *Tribolium castaneum* and the Evolution of Serial Homologs

**DOI:** 10.1534/g3.111.001537

**Published:** 2012-02-01

**Authors:** David R. Angelini, Frank W. Smith, Elizabeth L. Jockusch

**Affiliations:** * Department of Biology, American University, Washington, DC 20016-8007; † Department of Ecology & Evolutionary Biology, University of Connecticut, Storrs, CT 06269-3043

**Keywords:** appendage patterning, adult development, metamorphosis, developmental constraint, serial homology

## Abstract

Serial homologs are similar structures that develop at different positions within a body plan. These structures share some, but not all, aspects of developmental patterning, and their evolution is thought to be constrained by shared, pleiotropic gene functions. Here we describe the functions of 17 developmental genes during metamorphic development of the legs in the red flour beetle, *Tribolium castaneum*. This study provides informative comparisons between appendage development in *Drosophila melanogaster* and *T. castaneum*, between embryonic and adult development in *T. castaneum*, and between the development of serially homologous appendages. The leg gap genes *Distal-less* and *dachshund* are conserved in function. Notch signaling, the zinc-finger transcription factors related to *odd-skipped*, and *bric-à-brac* have conserved functions in promoting joint development. *homothorax* knockdown alters the identity of proximal leg segments but does not reduce growth. *Lim1* is required for intermediate leg development but not distal tarsus and pretarsus development as in *D. melanogaster*. Development of the tarsus requires *decapentaplegic*, *rotund*, *spineless*, *abrupt*, and *bric-à-brac* and the EGF ligand encoded by *Keren*. Metathoracic legs of *T. castaneum* have four tarsomeres, whereas other legs have five. Patterns of gene activity in the tarsus suggest that patterning in the middle of the tarsal region, not the proximal- or distal-most areas, is responsible for this difference in segment number. Through comparisons with other recent studies of *T. castaneum* appendage development, we test hypotheses for the modularity or interdependence of development during evolution of serial homologs.

Goethe (1790) and Owen (1848) recognized the importance of serial homology in plants and vertebrates, respectively, long before Darwin (1859) highlighted this phenomenon in support of evolution by natural selection. Anatomical duplication results in serial homologs, similar morphological structures that are repeated at different positions within an organism’s body plan, such as the appendages of arthropods. Serial homologs have widely divergent forms. Owen saw these “teleological modifications” as deviations (in his words, “adaptive masks”) of a group’s archetype, but Darwin claimed differences in serial homologs as evidence of adaptation through descent with modification. Among arthropods, modifications of the appendages, which form as extensions from the body, have generated structures adapted for sensation, feeding, and locomotion. More recently, duplication and diversification have emerged as crucial processes in evolution at the level of genes (Lynch and Force 2000) and genomes (Dehal and Boore 2005; Ohno 1970) as well as anatomy (Boxshall 2004; Snodgrass 1935).

Arthropod ventral appendages are organized with a proximal-to-distal (PD) axis with jointed segments. Among arthropods, ventral appendages share many aspects of developmental patterning (Abzhanov and Kaufman 2000; Angelini and Kaufman 2005b; Beermann *et al.* 2001; Inoue *et al.* 2002; Jockusch *et al.* 2000; Palopoli and Patel 1998; Panganiban *et al.* 1994; Prpic and Damen 2009; Rogers *et al.* 2002; Schoppmeier and Damen 2001). Nevertheless, appendage morphologies have diversified across body segments and species, and even in cases of conserved anatomy, divergent developmental genetic processes have been found (Angelini and Kaufman 2005a; Jockusch *et al.* 2000, 2004; Ronco *et al.* 2008; see also True and Haag 2001).

Here we evaluate two models for the evolution of developmental mechanisms controlling serially homologous appendages. The first model assumes that pleiotropic functions act as a strong constraint because mutations result in concerted changes in gene function that affect all appendage types (dependent model). The alternative model posits that pleiotropy is easily broken and that gene functions may evolve independently between appendages. These models represent ends of a spectrum and are not necessarily exclusive. A general understanding of the prevalence of dependent or independent evolution in the development of serial homologs is possible through a survey of developmental mechanisms in the appendages of multiple species. To test these hypotheses, we have examined the function of 17 candidate regulatory genes during metamorphosis in the legs of the red flour beetle, *Tribolium castaneum* (Table 1), and we consider these results in comparison with studies of other appendages in *T. castaneum* and the fruit fly *Drosophila melanogaster*.

**Table 1:** Candidate genes

Gene Name	Symbol	Protein Class	LG	GenBank	Clone Source
*decapentaplegic*	*dpp*	TGFβ ligand	4	NM_001039451	Sanchez-Salazar *et al.* 1996
*Distal-less*	*Dll*	homeobox TXF	7	NM_001039439	Jockusch *et al.* 2004
*dachshund*	*dac*	Ski/Sno-related TXF	4	XM_964678	Prpic *et al.* 2001
*homothorax*	*hth*	homeobox TXF	7	NM_001039400	Angelini and Kaufman 2004
*Lim1*		homeobox TXF	6	XM_964391	Angelini *et al.* 2009
*Notch*	*N*	Notch receptor	10	NM_001114381	*"*
*Serrate*	*Ser*	Delta/Serrate-type EGF	7	XM_964393	*"*
*Delta*	*Dl*	Delta/Serrate-type EGF	X	XM_964994	Current study
*odd-skipped*	*odd*	Zn-finger TXF	8	XM_966993	Angelini *et al.* 2009
*brother of odd with entrails limited*	*bowl*	Zn-finger TXF	8	XM_967045	*"*
*sister of odd and bowl*	*sob*	Zn-finger TXF	8	XM_966942	*"*
*drumstick*	*drm*	Zn-finger TXF	8	XM_966887	*"*
*Keren*	*Krn*	EGF ligand	3	XM_001813564	*"*
*bric-a-brac*	*bab*	BTB/Psq TXF	3	XM_001812888	*"*
*abrupt*	*ab*	BTB/Zn-finger TXF	5	XM_969854	*"*
*rotund*	*rn*	Zn-finger TXF	?	XM_966094	Current study
*spineless*	*ss*	bHLH/PAS TXF	10	XM_962783	Angelini *et al.* 2009

The chromosomal linkage group (LG) is listed, as well as the GenBank accession number of the known or predicted transcript. bHLH, basic helix-loop-helix; BTB, Bric-a-brac/Tramtrak/Broad complex domain; EGF, epidermal growth factor; PAS, Per/Arnt/Sim domain; Psq, pipsqueak; TXF, transcription factor.

Studies of leg development in the fruit fly *D. melanogaster* (reviewed by Angelini and Kaufman 2005b; Kojima 2004) provide a useful model for considering appendage development in other insect species and appendage types. In *D. melanogaster*, broad domains along the PD axis of the leg imaginal disc are established by gradients of secreted signaling molecules encoded by *wingless* and *decapentaplegic* (*dpp*) (Diaz-Benjumea *et al.* 1994; Lecuit and Cohen 1997; Wu and Cohen 1999). The distal “gap gene” in the leg disc is *Distal-less* (*Dll*) (Cohen and Jürgens 1989); *dachshund* (*dac*) is required for development of intermediate structures (Mardon *et al.* 1994); and proximal appendage development depends on the function of *homothorax* (*hth*) (Casares and Mann 2001). The transcription factors *bric-à-brac 1* and *bric-à-brac 2* (collectively *bab*) are encoded by paralogous, functionally redundant genes (Couderc *et al.* 2002) and are required for distal segmentation (Chu *et al.* 2002; Godt *et al.* 1993). The position of *bab* expression in the distal leg is influenced by repression from *odd-skipped* (*odd*) and its paralog *brother of odd with entrails limited* (*bowl*), which are expressed in the regions of the first (t1) and fifth tarsomeres (t5) (de Celis Ibeas and Bray 2003). Meanwhile, during the mid-third instar, other genes specific to distinct, presumptive PD regions become expressed in the leg disc, such as *spineless* (*ss*) and *rotund* (*rn*) from t2 to t4 (Duncan *et al.* 1998; St. Pierre *et al.* 2002). Epidermal growth factor (EGF) signaling is activated by *Dll* in cells giving rise to t5 and the pretarsus (Galindo *et al.* 2002). EGF activity is also key to activating genes responsible for pretarsus development, such as the homeobox transcription factor *Lim1* (Campbell 2005; Galindo *et al.* 2002; Kojima *et al.* 2005; Tsuji *et al.* 2000). The formation of joints is directed by Notch signaling and expression of the Notch ligands encoded by *Serrate* (*Ser*) and *Delta* results from the interaction of many of these genes (Bishop *et al.* 1999; de Celis *et al.* 1998; Greenberg and Hatini 2009; Rauskolb 2001; Rauskolb and Irvine 1999). These events pattern a leg with a single PD axis comprised of six primary segments (podomeres) separated by joints (the coxa, trochanter, femur, tibia, tarsus—which is subdivided into annuli called tarsomeres—and the pretarsus). This leg structure is conserved across insects.

In most Holometabola, adult appendages develop from fully functional external larval appendages, which are morphologically similar to the adult appendages (Svacha 1992; Tanaka and Truman 2005). Larval development is highly modified in Brachycera (Daly *et al.* 1998), such as *D. melanogaster*, meaning that some aspects of appendage morphogenesis in flies may not be representative of most insects. The appendages of *D. melanogaster* are suppressed in larval instars (Keilin 1915), and adult appendages develop from discs of presumptive imaginal tissue. Growth and patterning of these appendage primordia occurs internally during larval stages, and at metamorphosis the discs evert to form the adult appendages. Unlike the legless larvae of Brachycera, larvae of the tenebrionid beetle *Tribolium castaneum* have well-developed legs divided into five segments: coxa, trochanter, femur, tibiotarsus, and pretarsus (Figure 1B′; Sokoloff 1972). Metamorphosis involves an increase in leg size, segmentation within the tibiotarsus, and major changes in the shape and sensillae of appendages. Work in *Tenebrio molitor*, another tenebrionid beetle, has shown that the entire larval leg epidermis contributes to the adult leg epidermis. Each larval leg segment gives rise to the corresponding adult segment, with the exception that the larval trochanter gives rise to the proximal femur as well as the adult trochanter (Huet and Lenoir-Rousseaux 1976).

**Figure 1:**
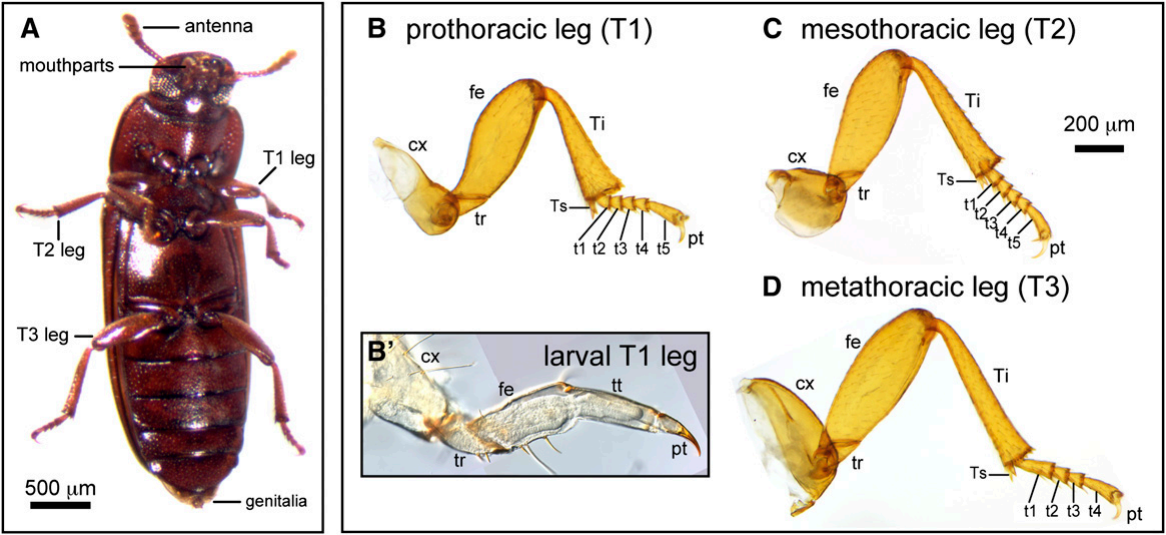
The ventral appendages of *Tribolium castaneum* include the antennae, mouthparts (mandibles, maxillae, and labium), thoracic legs, and genitalia. (A) Ventral view of an adult female. The thoracic legs of *T. castaneum* are roughly similar, with six true adult podomeres: coxa, trochanter, femur, tibia, tarsus, and pretarsus. The tarsus is subdivided by joints into tarsomeres, which lack independent muscles. The prothoracic leg (B) and mesothoracic leg (C) have five tarsomeres, whereas the metathoracic leg has four tarsomeres (D). The coxae of each type of leg have distinct shapes and sizes. (B′) Larval legs consist of five main segments: the coxa, trochanter, femur, tibiotarsus, and pretarsus. Abbreviations: cx, coxa; fe, femur; pt, pretarsus; t1-5, tarsomeres 1-5; T1–T3, thoracic segments 1-3; Ti, tibia; Ts, tibial spur; tt, tibiotarsus; tr, trochanter.

Comparative studies have found evidence for both conservation and divergence in aspects of appendage patterning across species and appendage types. For example, the “leg gap genes” have strongly conserved functions in embryonic and later development in many arthropods (*e.g.*, Angelini and Kaufman 2004; Moczek and Rose 2009; Schoppmeier and Damen 2001; Suzuki *et al.* 2009). However, gene functions in appendage allocation and PD axis specification have been found to vary (*e.g.*, Angelini and Kaufman 2005a; Jockusch *et al.* 2000; Ober and Jockusch 2006; Ronco *et al.* 2008).

*Tribolium castaneum* is a useful species for comparison with *D. melanogaster* because it is a holometabolous insect, like *Drosophila*, but the adult legs undergo development from larval legs. Variation in the number of tarsomeres in *T. castaneum* also makes it an attractive system. Pro- and mesothoracic legs have five tarsal elements (Figure 1, B and C), as do all legs of *D. melanogaster*, whereas the hindlegs of *T. castaneum* have four tarsomeres (Figure 1D). Compared with other leg traits, tarsomere number varies greatly among insects, but this heteromerous “5-5-4” tarsal pattern is characteristic of tenebrionoid beetles. A focus on metamorphic leg development also allows comparisons with previous studies of embryonic leg development in *T. castaneum* and other insects.

Our results illustrate a number of similarities between the development of the legs in *D. melanogaster* and *T. castaneum*. However, several important differences were found, including changes in the PD level of function for several genes as well as distinct gene functions in *T. castaneum*. We use these data, in combination with data on the metamorphic patterning of the antennae (Angelini *et al.* 2009) and mouthparts (Angelini *et al.* 2012) in *T. castaneum*, to examine the degree of modularity in the development of serial homologs and the implications this may have for evolution of appendage diversity.

## Materials and Methods

### General methods

Wild-type cultures of *Tribolium castaneum* were obtained from Carolina Biological Supply Company and reared using the supplier’s recommendations. The *Fused tarsi and antennae* (*Fta*) mutation of *Dll* was obtained from Susan J. Brown. The 17 candidate genes selected for study include transcription factors and components of conserved signaling networks (Table 1). Each gene has known roles in leg or antennal patterning in *D. melanogaster*. Cloned gene fragments were provided by colleagues as indicated by the references in Table 1, or cloned using standard methods that have been detailed elsewhere (Angelini *et al.* 2009). Additional genes were also cloned and tested functionally using RNAi; however, phenotypes in the legs were absent or not penetrant enough to be informative. These genes included *apterous* and *apterous-related*, *aristaless*, *clawless/C15*, *pdm/nubbin*, and *spalt*, which will not be described further in this study.

### Relative real-time polymerase chain reaction (PCR) determination of expression

The relative expression levels of genes in the tarsus and proximal leg were determined using real-time PCR. The pro- and mesothoracic legs of approximately 20 pupae were bisected at the tibia−tarsus joint to generate separate pools of proximal and distal tissue for RNA extraction using Trizol (Invitrogen/Life Technologies) followed by DNase I treatment to remove genomic DNA. The iScript cDNA Synthesis Kit (BioRad) was used for first-strand cDNA synthesis. Amplification of target cDNAs used a SYBR Green realtime PCR kit (Absolute Scientific) on a BioRad iCycler instrument. Calculations of relative target sequence abundance were normalized with *18S* ribosomal RNA and adjusted for measured primer efficiency (Pfaffl 2001). All primer sequences are available from the authors upon request.

### RNA interference

Knockdown phenotypes were generated in adult beetles using RNA interference (RNAi). Prepupal larvae were injected with double-stranded (ds) RNA following the methods of previous studies (Angelini *et al.* 2009, 2012; Tomoyasu and Denell 2004). *GFP* dsRNA was used as a control to determine the rates of spontaneous or injection-induced malformations. Because of the potential for functional redundancy of some genes studied here, we also included some treatments in which multiple genes were targeted by injecting multiple dsRNAs (Table 2). Validation of RNAi knockdown was performed by real-time PCR comparison of target gene expression in control (*GFP*) dsRNA and gene-specific dsRNA treatments (Table 2), as described in an accompanying article in Genetics, Angelini *et al.* (2012; see also Aspiras *et al.* 2011).

**Table 2:** Summary of RNA inference effects

			Phenotypic Penetrance					
dsRNA Sequence	dsRNA Size, bp	Target Gene Knockdown*^a^*	Number Scored	Legs				Defects in Other Appendages
				Unaffected	Mild	Moderate	Severe	
*GFP*	600		83	96.4%	2.4%*^b^*	0	1.2%*^b^*	1.2%*^b^*
*dpp*	812	66% ± 13%*	35	37%	17%	17%	29%	40%
*Dll*	462	75% ± 4.9%*	42	17%	4.8%	17%	62%	91%
*dac*	359	30% ± 16%	66	38%	11%	23%	29%	76%
*hth*	335	44% ± 7.5%*	205	34%	39%	22%	6%	90%
*Lim1*	388	56% ± 31%	16	31%	6.3%	25%	38%	75%
*Notch*	409	24% ± 9.0%*	31	23%	6.5%	19%	52%	94%
*Ser*	329	n/a*^c^*	151	14%	0.7%	2.6%	83%	97%
*Delta*	180	71% ± 14%*	75	85%	6.7%	6.7%	1.3%	38%
*Ser*, *Delta*	—		23	44%	0	4.3%	52%	78%
*odd*	211	65% ± 7.3%*	50	36%	14%	8.0%	42%	86%
*bowl*	342	63% ± 5.2%*	57	46%	8.8%	8.8%	37%	79%
*sob*	321	61% ± 8.0%*	16	6.3%	0	56%	38%	94%
*drm*	225	n/a*^c^*	30	3.3%	0	3.3%	93%	100%
*odd*, *bowl*, *sob*	—		16	50%	6.3%	19%	25%	81%
*odd*, *bowl*, *sob*, *drm*	—		37	5.4%	5.4%	0	89%	100%
*Krn*	188	67% ± 9.2%*	19	11%	5.3%	21%	63%	90%
*bab*	774	n/a*^c^*	22	18%	4.5%	9.1%	68%	68%
*ab*	198	42% ± 6.3%*	31	26%	6.5%	0	68%	81%
*rn*	127	78% ± 9.0%*	52	75%	2%	14%	10%	21%
*ss*	355	n/a*^c^*	19	58%	16%	5.3%	21%	100%
total			1076	33%				76%

The level of target gene knockdown was determined by real-time PCR comparisons of pooled pupae to nonspecific *GFP* dsRNA controls. The phenotypic effects on legs and other appendages were scored after metamorphosis.

* Significant difference from gene expression in *GFP* control specimens (Welch’s t-test, *P* < 0.05).

a 0% represents no reduction in activity, while 100% is complete suppression.

b One control specimen eclosed with truncated T1 legs (scored as severe). Two specimens were missing a single tarsal joint. One specimen had a fusion of the distal- most segments of one antenna.

c Suitable primers for this gene were unavailable (i.e., n/a).

Adult morphology was examined after clearing overnight in a solution of 20% glycerol in glacial acetic acid at 50° (modified from van der Meer 1977). Pharate or eclosed adults (n = 1076) were scored for up to 64 anatomical characters related to the legs, including the presence or absence of each appendage segment, degree of fusion between segments, and changes in size, shape, and characteristic bristle patterns. This data matrix (supporting information, File S1) was used to quantify the penetrance of RNAi for each gene in each leg structure and to assess the severity of phenotypes.

### Microscopy and imaging

Photomicrographs of dissected appendages were obtained with an Olympus digital camera on a Zeiss Axioskop compound microscope. For electron microscopy, specimens were prepared by overnight dehydration in ethanol, followed by a 15-min immersion in hexamethyldisilazane. Specimens were then sputter coated in gold palladium and imaged with a Zeiss DSM982 Gemini field emission scanning electron microscope.

## Results

On the basis of the development of *D. melanogaster*, candidate genes were identified for study in *T. castaneum* (Table 1). Expression and functional studies were conducted with the resulting group of 17 genes in *T. castaneum*. Real-time PCR confirmed that RNAi resulted in a reduction in the expression of target genes, with knockdown levels ranging from 24% to 78% (mean of 57% reduction) in pooled pupal samples (Table 2). RNAi phenotypes were qualitatively consistent within dsRNA treatments, and for many genes, these phenotypes could be ordered into a series from mild to severe, resembling a hypomorphic mutant series. RNAi was highly penetrant, with an average of 84% of individuals having defects in at least one appendage. Penetrance in the legs averaged 67% (Table 2) and did not vary significantly between thoracic segments; in general, affected individuals showed similar defects in multiple legs. Sample sizes of individuals scored for leg defects ranged among treatments from 16 to 205 (mean = 48; Table 2). The RNAi phenotypes are described under the subheadings to follow, arranged by the developmental processes affected.

### Proximal-to-distal gene expression bias in the leg

**Figure 2:**
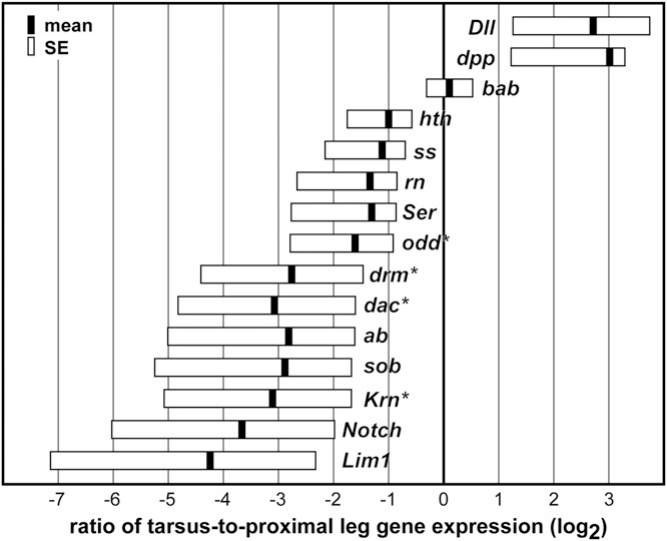
Relative expression of candidate genes is compared in the tarsus and proximal leg of pupae. Expression ratios are given in log_2_ scale. Zero represents equal expression in each region; genes appearing to the right are enriched in the tarsus, relative to the rest of the leg. Black bars indicate the mean expression ratio, whereas boxes indicate standard error. Genes with expression ratios significantly different from 1 (equal expression in both regions) are denoted with an asterisk (Welch’s *t*-test, *P* < 0.05).

The relative PD expression bias for genes was determined in the pupal leg using relative real-time PCR (Figure 2). Expression of several genes was significantly enriched in either the tarsus or proximal leg at the pupal stage. For most genes, regions of expression-bias correlated well with regions affected by RNAi. Two genes, *Dll* and *dpp*, whose depletion produced phenotypes in the tarsus, showed significantly elevated expression in that tissue. Notch signaling components (*Notch* and *Ser*), which affected the entire leg, and genes with RNAi phenotypes in the tibia (*dac*, *Lim1*, and *Krn*) had a significant proximal bias in expression. We detected an unexpected proximal bias in expression for three genes with depletion phenotypes in the tarsus (*rn*, *ss*, and *abrupt*); however, the expression ratio for these genes was not significantly different from 1 (Welch’s *t*-test, *P* > 0.05).

### Genes required for growth of large leg regions

**Figure 3:**
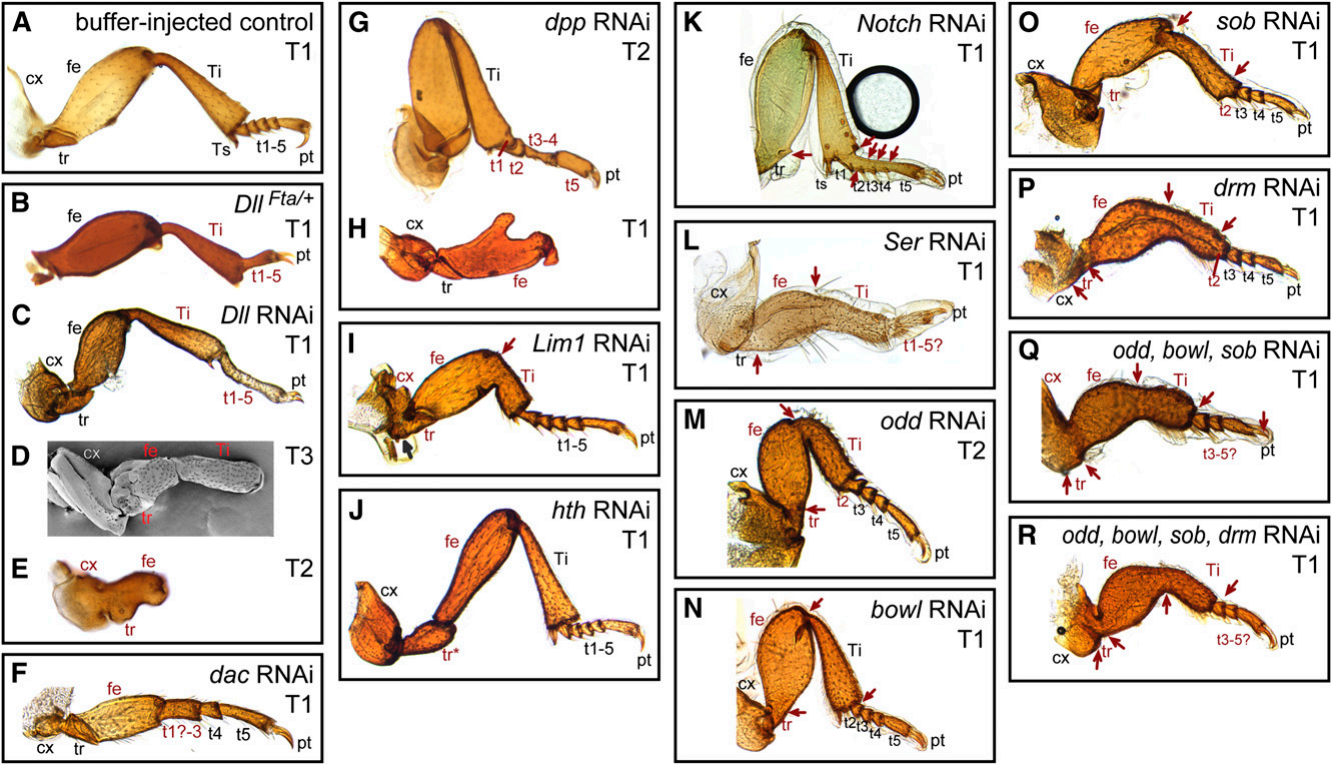
RNA interference effects on adult legs. (A) Control treatments were indistinguishable from unmanipulated beetles. (B) *Dll^Fta^* heterozygotes develop with reduced tarsi, lacking joints. (C) This phenotype is similar to mild *Dll* RNAi specimens. (D) *Dll* RNAi also resulted in stronger phenotypes, in which structures distal to the tibia are deleted. (E) In the most severe *Dll*-depleted individuals, the legs are truncated within the femur, which is reduced. Joints are also absent from the remaining segments. (F) Severe *dac* RNAi specimens had deletions of the distal femur, tibia, and proximal tarsomeres. (G) RNAi targeting *dpp* caused alterations of the proximal tarsomeres in mildly affected specimens. (H) In more severe *dpp* RNAi specimens, the legs are truncated in the mid-femur. (I) *Lim1* RNAi caused the loss of proximal leg joints (red arrow) and a reduction of the distal femur and proximal tibia. Although the femur−tibia joint did not form normally, an anatomical boundary was present at this position. (J) Knockdown of *hth* caused a homeotic transformation of the coxa, trochanter and proximal femur toward more distal morphologies. (K) *Notch* RNAi eliminated most joints from the leg (red arrows). (L) *Ser* RNAi eliminated joints (red arrows) and reduced the overall length of the leg. (M-R) Depletion of *odd*-related genes caused reduction of the leg and loss of proximal joints (red arrows), as well as deletion of proximal tarsomeres. Abbreviations: cx, coxa; fe, femur; pt, pretarsus; t1-5, tarsomeres 1-5; T1, prothoracic leg; T2, mesothoracic leg; T3, metathoracic leg; Ti, tibia; tr, trochanter; Ts, tibial spur. Structures with a defect are labeled in red.

**Figure 4:**
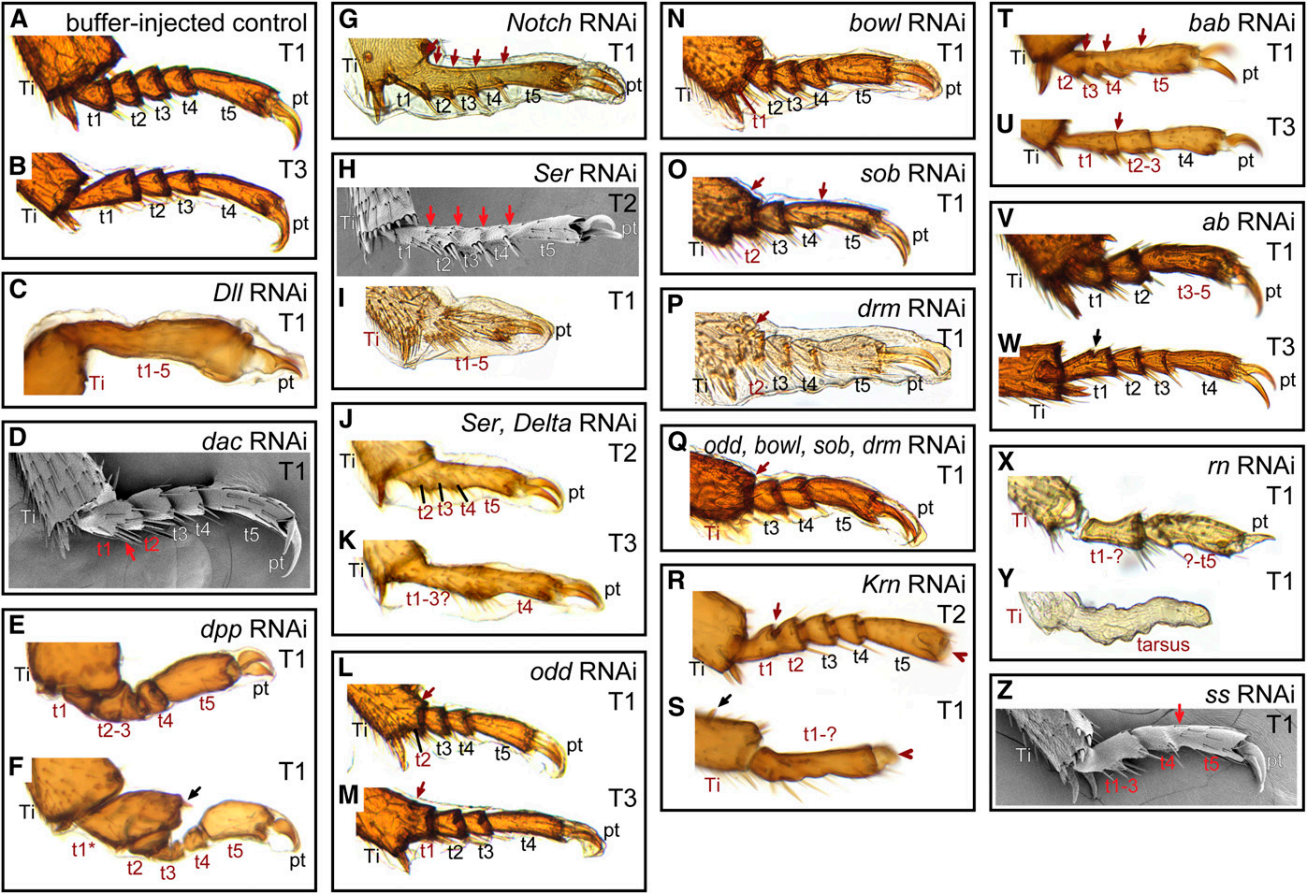
Phenotypes in the tarsus produced through RNAi. (A) Normal tarsus anatomy in the pro- and mesothoracic legs includes five tarsomeres. The distal tibia is also visible, showing the distal tibial spurs. (B) The metathoracic legs normally have four tarsomeres. (C) *Dll* RNAi produces mild specimens in which the tarsomeres lack joints and the region is reduced. (D) Depletion of *dac* produced fusions in the proximal tarsus. (E) Knockdown of *dpp* reduced t1-t4. (F) Rarely *dpp* RNAi caused a transformation of the first tarsomere (t1^*^) toward a distal tibia identity, as indicated by an ectopic tibial spur (black arrow). (G) *Notch* RNAi caused the loss of joints in the tarsus (red arrows). (H) Mild *Ser*-depleted specimens also lacked joints between tarsomeres (red arrows). (I) Moderate *Ser* knockdown phenotypes included fusion and reduction of the tarsus. (J–K) Simultaneous depletion of *Ser* and *Delta* produced phenotypes similar to *Ser* RNAi alone. (L—Q) RNAi targeting the *odd*-related genes caused fusion of proximal tarsomeres with the tibia (L, M, O), or adjacent tarsomeres (O). The first tarsomere (L, N, P) and sometimes also the second (Q) were deleted in moderate and severe specimens. (R) *Krn* RNAi eliminated the pretarsus (red arrowhead), and caused occasional loss of joints (red arrow) in mild specimens. (S) More severely affected *Krn* RNAi specimens had a reduction of the tarsus with complete loss of joints. Tibial spurs were also deleted, although ectopic tibial spurs could sometimes be found (black arrow). (T–U) Knockdown of *bab* caused loss of joints in the tarsus (red arrows) and fusion of tarsomeres. (V) Depletion of *ab* caused fusions of tarsomeres. (W) Rarely partial ectopic joint formation was observed in *ab* RNAi (black arrow). (X) RNAi targeting *rn* caused a reduction of the tarsus and failure of joint formation. (Y) In severely affected *rn* RNAi specimens the pretarsus also failed to form. (Z) Knockdown of *ss* also caused fusions of tarsomeres (red arrow). Abbreviations are as in Figure 3.

Two of the classic leg “gap genes,” *Distal-less* (*Dll*) and *dachshund* (*dac*), were required for the specification and growth of large regions of the developing metamorphic leg in *T. castaneum*. Similar RNA interference results for these genes have been reported by Suzuki *et al.* (2009), and we expand the anatomical description of phenotypes here. RNAi targeting *Dll* produced a range of phenotypes. In the mildest phenotypes, the tarsus lacked joints and the tarsus, tibia, and femur were shorter than in wild type (Figures 3C and 4C). Hypomorphic alleles of *Dll* have been isolated in *T. castaneum* (Beermann *et al.* 2001); their phenotypes closely resemble this mild *Dll* RNAi effect (Figure 3, B and C). More severely affected *Dll* RNAi specimens had legs truncated at the level of the tibia (Figure 3D) or femur (Figure 3E). In the most severely affected individuals, the joints between proximal segments were also incomplete (Figure 3E). Depletion of *dac* resulted in loss of an intermediate portion of the legs, extending from the middle of the femur to the proximal tarsus (Figure 3F). The joint at the distal end of the femur was abnormal and did not resemble either the wild-type femur−tibia or tibia−tarsus joint. The proximal tarsal elements were also affected, with tarsomere 1 (t1) being deleted or fused to t2 (Figure 4D). RNAi depletion of each of these genes closely paralleled mutant phenotypes from *D. melanogaster* (Cohen and Jürgens 1989; Mardon *et al.* 1994).

RNAi disruption of *dpp* activity in *T. castaneum* prepupae also produced some adults (5 of 35 scored) with large deletion phenotypes (Figure 3H), although most specimens had defects restricted to the tarsus (Figures 3G and 4E). Truncations occurred in the middle of the femur, with loss of more distal podomeres. The coxa and trochanter of these legs appeared normal; however, the femur was malformed and its distal end was often bifurcated into two prongs (Figure 3H). This *dpp* RNAi phenotype was dramatically asymmetrical (which was unusual for the RNAi phenotypes of most genes we investigated) and all individuals with one or more truncated legs also had multiple anatomically normal legs. This asymmetry was also observed with milder *dpp* RNAi effects in the tarsus. Truncation or bifurcation of the leg was also found after clonal disruption of Dpp signaling in the fly leg (Theisen *et al.* 1996). In the tarsus, *dpp* depletion caused reduction and malformation of t1–t4 (Figures 3G and 4, E and F). The pretarsus was not affected.

RNAi targeting *Lim1* in *T. castaneum* prepupae caused a reduction of the intermediate region of the leg (Figure 3I), which we interpret as a partial deletion of the distal femur and proximal tibia. Shape and bristle patterns indicate that the proximal femur and distal tibia developed normally in *Lim1* RNAi. The region between these podomeres, where the presumptive femur-tibia joint normally forms, was sharply angled and constricted ventrally, suggesting that some differentiation of separate podomeres still occurred. The intervening joint was lost or malformed (8 of 16 specimens scored). Loss of the coxa-trochanter joint was also observed in severely affected specimens (3 of 16 scored). In *D. melanogaster*, *Lim1* is expressed in four discrete domains along the PD axis (Campbell 2005; Lilly *et al.* 1999; Tsuji *et al.* 2000), and RNAi phenotypes in *T. castaneum* closely parallel those in the proximal leg of *D. melanogaster Lim1* mutants, in which the coxa is deformed or absent and the femur is greatly reduced and fused to an abnormally bent tibia (Pueyo *et al.* 2000; Tsuji *et al.* 2000). The pretarsus in the legs of *D. melanogaster* mutants is also absent (Pueyo *et al.* 2000; Tsuji *et al.* 2000), whereas in all *T. castaneum Lim1* RNAi specimens the tarsus and pretarsus were unaffected.

Overall, these phenotypes reveal that appendage regions which are already present in the *T. castaneum* larva can be deleted at metamorphosis. These results indicate an ongoing requirement for *Dll*, *dac*, *dpp*, and *Lim1* in maintenance and adult development of specific leg regions.

### Homeotic transformation of the proximal leg as a result of *homothorax* RNAi

Among the genes examined in this study, only *homothorax* depletion produced a homeotic phenotype. The proximal region of the leg imaginal disc of *D. melanogaster* is specified in part by *homothorax* (*hth*), which encodes a homeobox transcription factor (Wu and Cohen 1999). Leg imaginal discs lacking *hth* develop a normal tarsus but have a single reduced, proximal segment with identity from more than one of the missing podomeres (Casares and Mann 2001). Depletion of *hth* in *T. castaneum* prepupae also caused defects in the proximal adult legs (Figure 3J). Unlike the reduced phenotype of the *D. melanogaster hth* null legs, *hth*-depleted *T. castaneum* had fully elongated legs with the normal number of leg segments. However, the morphology of the three proximal podomeres was altered. The most obvious effects were in the coxa and trochanter, which were enlarged and had bristle patterns more like those of more distal leg segments. The presumptive femur was narrower but similar in length to the wild-type femur. Joints between the coxa, trochanter, and femur were present, but resembled the wild-type femur–tibia joint in morphology. The prothoracic coxa was more rounded in shape than in wild type. This may represent either a transformation of the coxa toward distal identity, or it may represent a transformation of the prothoracic coxa toward the identity of a mesothroracic coxa (compare Figures 1, B and C, and 3J). However, the placement of the coxa-trochanter joint differed between the transformed prothoracic and normal mesothoracic coxae. The distal leg was unaffected. Taken together, these phenotypes suggest that depletion of *hth* removes the normal identity cues in the proximal leg, but does not affect growth along the PD axis or the location and formation of joints. The resulting podomeres have the shape and bristle pattern suggestive of the femur, but their identity remains ambiguous. One hypothesis is that these segments have a mixed femur/tibia identity, as in *D. melanogaster hth* loss-of-function (Casares and Mann 2001).

### The Notch signaling pathway and *odd-skipped* paralogs are require for both leg growth and joint formation

Notch signaling is required early for elongation of the PD axis in *D. melanogaster* leg discs, as well as for joint formation later in development (Bishop *et al.* 1999; de Celis *et al.* 1998; Rauskolb and Irvine 1999). *Notch*-mediated joint formation has been proposed as a defining characteristic of arthropods (Prpic and Damen 2009). In *T. castaneum*, we examined depletion phenotypes for *Notch* as well as its ligands, encoded by *Serrate* (*Ser*) and *Delta*, and found evidence that both the axis elongation and joint formation roles are conserved.

Knockdown of *Notch* or *Ser* blocked joint formation in the legs (Figure 3, K−L). *Notch* RNAi individuals had legs that were relatively normal in size and shape but that lacked most joints (Figure 3K). Joint loss phenotypes were much less penetrant in the femur–tibia (26 of 186) and tarsus–pretarsus (10 of 186) joints than they were in the other joints between the primary segments (18%–60% joint loss). Joints between the tarsomeres were especially sensitive to depletion of *Notch* (Figure 4G), and in the mildest phenotypes, joint loss only occurred between tarsomeres. Despite the absence of joints, differentiation of individual tarsomeres was suggested by the retention of the distal bristles present on t1–t4 (on pro- and mesothoracic legs—or t1–t3 on metathoracic legs) which were spaced as in wild-type individuals. *Delta* RNAi was far less penetrant (11 of 75 *Delta*-depleted specimens had leg defects *vs.* 28 of 31 *Notch*-depleted specimens) despite significant reduction in *Delta* transcripts after RNAi (71% reduction ±14%). *Delta*-depleted specimens with phenotypes in the legs had a failure of joint formation. These individuals lacked all joints in the tarsus (8 of 443 legs) or joints between coxa, trochanter, and femur (10 of 444 legs) or both of these states (1 of 443 legs). *Ser* RNAi phenotypes were highly penetrant and typically severe (Table 2), including loss of joints and reduction of the legs overall. The pretarsus was only rarely affected (5% of specimens). RNAi targeting *Ser* (but not *Notch* or *Delta*) resulted in drastic reduction in the length of legs (Figure 3L), with the tarsus and tibia more severely reduced compared with more proximal segments. The structures most affected by *Ser* RNAi are also those that undergo the greatest increase in size during normal metamorphosis (compare Figure 1, B and B′). Mild *Ser* RNAi phenotypes lacked tarsal joints and showed fusions of tarsomeres (Figure 4, H and I). Despite penetrant phenotypes in the tibia and tarsus, the pretarsus and tibial spurs were not strongly affected by depletion of any Notch signaling component, having been deleted in only 3.6% (61/1679) or 4.8% (76/1572) of legs scored, respectively, in all Notch signaling RNAi experiments.

Depletion of *Notch*, *Ser*, and *Delta* had different effects on bristle development. In *Notch* RNAi individuals, most bristles were absent, although the large pegs at the end of each tarsomere remained (Figure 3K). In contrast, many bristles remained after *Ser* RNAi, including the regularly arranged pattern of bristles on the femur and tibia (Figure 3L). With the exception of this effect on bristles, depletion of *Notch* had less severe effects on leg development than depletion of *Ser*. The Ser ligand acts redundantly with Delta in some contexts in *D. melanogaster* (Zeng *et al.* 1998). Therefore, it would be predicted that *Notch* RNAi phenotypes should be more severe than those of *Ser*. However, it is possible that severe *Notch* depletion is lethal and that only relatively mild phenotypes were recovered here. Simultaneous depletion of *Ser* and *Delta* produced phenotypes similar to *Ser* RNAi alone, with reduction of leg length, loss of joints, and fusions of tarsomeres (Figure 4, J and K).

Genes related to *D. melanogaster odd-skipped* (*odd*) also affected large regions of the legs in *T. castaneum*. This gene family includes four linked zinc-finger transcription factors: *odd*, *brother of odd with entrails limited* (*bowl*), *sister of odd and bowl* (*sob*), and *drumstick* (*drm*). The similarity of *odd*-paralog and *Ser* RNAi phenotypes suggests that these genes lie in the same pathway in *T. castaneum* as they do during leg development in *D. melanogaster*. In the leg imaginal disc of *D. melanogaster*, *odd*-related genes are required for proper patterning of the tarsus (de Celis Ibeas and Bray 2003; Hao *et al.* 2003), where they may act to stabilize Notch signaling (Greenberg and Hatini 2009). Three of these genes (*odd*, *bowl*, *sob*) share substantial sequence similarity in the regions we targeted for knockdown in *T. castaneum*; therefore, we do not distinguish specific roles for each of these genes. Phenotypes for these single-gene knockdowns were similar to one another and to phenotypes resulting from simultaneous targeting of all four paralogs.

The legs of *T. castaneum* were reduced in length after knockdown of *odd* paralogs, with most of the reduction occurring in the femur and tibia (Figure 3, M−2R). The shape changes in these segments also resembled the shape changes observed in response to *Ser* RNAi (Figure 3L). In the most extreme phenotypes, the tibia was broadest centrally, with rounded lateral edges. Joints between the trochanter, femur, tibia, and tarsus also frequently failed to form. Unlike the joint loss found with RNAi targeting Notch signaling, which affected all tarsomeres equally, proximal tarsal elements (t1–t2) were far more sensitive to *odd*-paralog depletion than were more distal tarsal elements. Fusion occurred in 39% of t1–t2 joints *vs.* 10% to 13% of more distal tarsomere joints (for scored T1 and T2 legs; *e.g.*, Figure 4O). In mildly affected RNAi specimens, the only defects we observed were in the tarsus, where t1 was deleted or fused to the distal tibia (Figure 4, M, N, and P). Occasionally the second tarsomere was also deleted or fused to the distal tibia (Figure 4, L and O−Q). Often, loss of the proximal tarsomere was accompanied by deletion of the spurs at the distal end of the tibia (333 of 874 legs scored, *e.g.*, Figure 4, O and Q).

### Genes required primarily in the tarsus and pretarsus

Depletion of several genes yielded phenotypes that were restricted to the tarsus or pretarsus. In *D. melanogaster*, several EGF ligands are secreted from the distal appendage tip and loss of EGF signaling leads to the loss of t5 and the pretarsus (Clifford and Schupbach 1989; Galindo *et al.* 2002) with rare reduction of the entire tarsus and pretarsus to a single tarsomere-like structure (Campbell 2002). The *T. castaneum* genome has only one activating EGF ligand, with greatest sequence similarity to *Krn* (*Tribolium* Genome Sequencing Consortium 2008). Knockdown of *Krn* in *T. castaneum* led to dramatic defects in the antennae and mouthparts (Angelini *et al.* 2009; see also accompanying article in Genetics, Angelini *et al.* 2012). However, in the legs, *Krn* RNAi phenotypes were restricted to the distal leg (Figure 4, R and S). The most common phenotype was loss of the pretarsus (17 of 19 scored). In more strongly affected individuals, tarsomeres were also fused to one another (Figure 4R), and in the most severely affected individuals, the entire tarsal region was reduced and lacked joints (Figure 4S). In moderately and severely affected specimens the distal tibia, near the tibia−tarsus joint, was also abnormally shaped, with absent or misplaced tibial spurs (Figure 4S, red arrow).

Downstream of EGF signaling in *D. melanogaster* the pretarsus and distal tarsomeres are patterned through the activity of a feed-forward gene circuit that includes *Lim1*. Loss of *Lim1* function results in deletion of the claw and reduction or fusion of t4–t5 (Campbell 2005; Pueyo *et al.* 2000; Tsuji *et al.* 2000). As described previously, *Lim1* RNAi caused deletions spanning the femur-tibia joint and failure of joint formation in the proximal leg in *T. castaneum*. Defects in the tarsus were rare and consisted of the incomplete fusion of adjacent tarsomeres (2 of 16 specimens; this rate is not significantly different from that seen in *GFP* RNAi, Fisher’s exact test, *P* = 0.18).

Depletion of *dpp* also produced tarsal defects that were pronounced in the proximal tarsus (Figure 4, E and F), in addition to defects in the more proximal leg segments (Figure 3H). Defects included fusions and reduction of tarsomeres, but these defects were qualitatively different from reduction and fusion occurring with other dsRNA treatments. Tarsomeres were typically reduced in width, not length (Figure 4F, t3-t4). In two specimens, the proximal tarsomere was enlarged and bore the spurs indicative of tibial identity (Figure 4F, t1^*^).

Depletion of two BTB-class transcription factors, *bric-à-brac* (*bab*) and *abrupt* (*ab*), produced tarsal defects with high penetrance. In *D. melanogaster* redundant *bab* paralogs are required for the proper development of the distal tarsomeres and mutations in *bab* affect t2-t5, causing distal deletions, fusions, or transformation to more proximal identity (Couderc *et al.* 2002; Godt *et al.* 1993). RNA interference targeting the single *bab* ortholog in *T. castaneum* caused a similar phenotype wherein tarsomeres were reduced and fused (Figure 4, T and U). In contrast to *bab* phenotypes in *D. melanogaster*, *T. castaneum bab* RNAi phenotypes often included t1 in fusions (91 of 104 legs scored). Depletion of *ab* also caused the loss of joints, fusion of adjacent segments, and/or a small deletion in the tarsal region (Figure 4, V and W), but in a different pattern than *bab* depletion. Loss of joints or fusions of tarsomeres 1-2 and 3-5 (or 3-4 in the metathoracic tarsi) occurred in 37 of 42 *ab* RNAi specimen legs, with the remainder being wild type or missing only a single joint. Rarely, *ab* knockdown individuals were recovered with a partial ectopic joint and accompanying ventral macrochetes in the metathoracic tarsus (2 of 57 T3 legs scored; Figure 4W, arrowhead). In *D. melanogaster*, *abrupt* mutants have altered legs, with distal regions more strongly affected or deleted (Hu *et al.* 1995); the mutant phenotype has not been described in detail.

RNAi targeting two other transcription factors produced leg defects that were limited to the tarsal region with relatively low penetrance: *rotund* (*rn*) and *spineless* (*ss*). In *D. melanogaster*, *rn* and *ss* are transiently expressed in an intermediate region of the tarsus, and t2-t4 are lost in response to mutations in either gene (Cavener *et al.* 1986; Duncan *et al.* 1998; Kozu *et al.* 2006; St. Pierre *et al.* 2002). Depletion of each of these genes in *T. castaneum* also caused the loss of joints, fusion of adjacent tarsomeres, and reduction of the tarsal region (Figure 4, X−Z). Knockdown of *rn* also frequently produced fusion between the tarsus and pretarsus accompanied by abnormal development of the pretarsus (38 of 77 legs in specimens with *rn*-depletion defects; Figure 4, X and Y). Shippy *et al.* (2009) reported similar *ss* RNAi phenotypes in making the case that the classic *T. castaneum antennapedia* mutations are allelic to *ss*. The strongest of these *ss* alleles cause reductions in the tarsus resembling *ss* RNAi phenotypes (Shippy *et al.* 2009).

## Discussion

**Figure 5:**
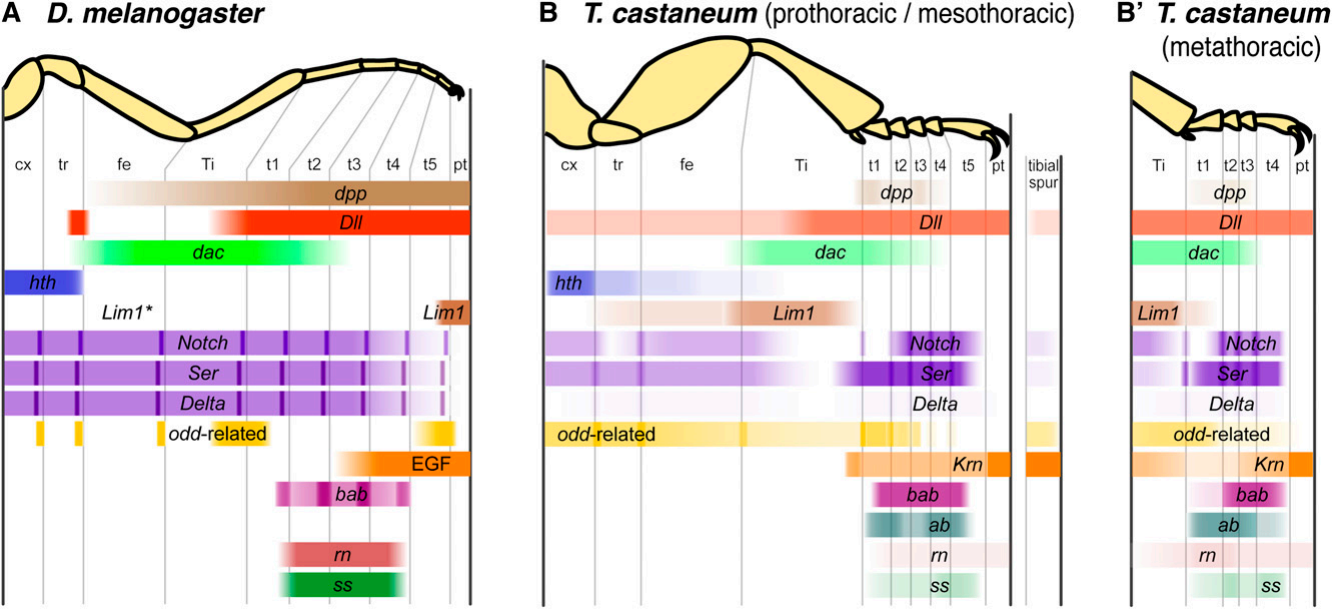
Summary of RNAi phenotypes in the legs. (A) Functional domains of genes in the leg of *D. melanogaster*. For each gene, colored bars represent the region on the corresponding adult leg in which the gene is expressed and/or functions, focusing on the late third instar larval imaginal leg disc. *Dll*, EGF, and *dpp* are also required in the embryo for initiation of imaginal leg disc primordia. *odd*-related genes are expressed more broadly in the tarsus earlier in development, and are required for proper formation of tarsomere joints. In *D. melanogaster*, *ab* has been observed in the leg discs, but its expression pattern has not been described in detail (Hu *et al.* 1995). ^*^Lim1 expression has also been detected in multiple domains in the proximal imaginal leg disc, including the femur (Tsuji *et al.* 2000). (B) Patterning of the pro- and mesothoracic legs of *T. castaneum*. Intensity of expression indicates phenotypic penetrance of RNAi effects. The tibial spur is represented by the column at right. (B′) The metathoracic leg has only four tarsomeres, and its patterning of the intermediate tarsomeres (t2-t3) differs from the more anterior legs.

To explore how distinct appendage types are patterned, we have examined the function of 17 genes during metamorphic appendage development of the red flour beetle *Tribolium castaneum*. The majority of the genes studied here have conserved roles in leg patterning in *D. melanogaster* and *T. castaneum* (summarized in Figure 5), but several have differences in their area of functional effect; these differences range from slight to dramatic. After comparing leg patterning in *Tribolium* and *Drosophila* and across developmental stages within *Tribolium*, we conclude by using the genes with divergent functions to test conflicting predictions about how the appendage patterning networks of serial homologs evolve.

### Patterning of the primary leg segments

All insects share a conserved leg morphology consisting of six primary segments, including an annulated tarsus. For many genes in this study (although not all) gene functions in the legs appear to be very similar in *T. castaneum* and *D. melanogaster*, as expected if an ancestral leg patterning network has been conserved in both lineages. Genes involved in the outgrowth of appendages, such as *Dll*, EGF, and Notch signaling components, are conserved in that role in legs (Figures 3, D, E, and L and 4, I and T), as well as in other appendage types. Similarly, Notch signaling is conserved it its role in joint formation in both insects. Loss of function of *Notch* or one of its ligands causes a failure of appendage joint development (Figures 3, K and L and 4, G−K). On the basis of studies of Notch signaling in a spider, it has recently been proposed that Notch-mediated cuticular joint formation is a synapomorphy of arthropods, which may have contributed to their success and diversification (Prpic and Damen 2009).

The *odd-skipped* family of zinc-finger transcription factors is an important regulator of leg development; these proteins interact with Notch signaling to specify joints in the primary leg segments. In *D. melanogaster*, all four family members are expressed at the joints between primary leg segments, where they function in joint formation. *bowl* is more broadly expressed in the tarsus, where loss of function leads to loss of joints, as well as reduction and other patterning defects (de Celis Ibeas and Bray 2003; Hao *et al.* 2003). In *T. castaneum*, *odd*-related genes are also required for tarsal development, as well as for elongation and joint formation in more proximal leg segments, as shown by the RNAi phenotypes (Figures 3, M−R and 4, L−Q). Considering the complex patterns of expression and function in these genes, their conservation is noteworthy.

Another group of genes with largely conserved functions is the “limb gap genes.” *Distal-less, dac,* and *hth* specify broad domains of identity along the leg PD axis in *D. melanogaster*, *T. castaneum*, and other species. The loss of function phenotypes for *Dll and dac* result in deletion of the distal and intermediate domains, respectively (Cohen and Jürgens 1989; Mardon *et al.* 1994; Suzuki *et al.* 2009; Figure 3, B−F). Similar RNAi phenotypes have also been reported for these genes in the beetles *Harmonia axyridis* (*Dll*: Niimi *et al.* 2005) and *Onthophagus taurus* (*Dll*, *dac*, *hth*: Moczek and Rose 2009), the milkweed bug *Oncopeltus fasciatus* (*Dll*, *dac*, *hth*: Angelini and Kaufman 2004), and the spider *Cupiennius salei* (*Dll*: Schoppmeier and Damen 2001). In both *D. melanogaster* (Casares and Mann 2001) and *T. castaneum*, *hth* is required for proper development of the proximal most leg segments. However, aspects of the *hth* phenotypes differ in significant ways between these species; in particular, in *D. melanogaster*, loss of *hth* expression leads to a reduction in the number of podomeres, while in *T. castaneum*, all podomeres are retained, although their identities are altered.

The role of *dpp* in leg development has been an important test case for understanding the conservation of developmental systems. In the leg imaginal disc of *D. melanogaster*, *dpp* is required for dorsal-ventral axis formation, and null clones lacking one of the Dpp receptors (encoded by *thickveins* and *punt*) lead to truncations and bifurcations of the limb axis (Theisen *et al.* 1996). This is similar to the severe phenotype obtained here for *dpp* RNAi in adult legs (Figure 3H). The role of *dpp* in proximodistal patterning of embryonic appendages in *T. castaneum* is unclear. In mild embryonic RNAi phenotypes, *T. castaneum* limb buds develop normally (Ober and Jockusch 2006), while in stronger phenotypes, early embryonic dorsoventral axis patterning is greatly altered, and appendages fail to form (van der Zee *et al.* 2006).

### Tarsal development and evolution

It is interesting that the basic developmental mechanisms responsible for overall leg patterning (*i.e.*, differentiation of the PD axis into the six true segments) and for tarsal patterning (subdivision of the tarsus into jointed tarsomeres) are fundamentally similar, whereas the evolutionary diversity of these traits is very different. The pattern of subdivision of the PD axis of the leg differs between arthropod classes, but it has remained fixed within insects for 396 million years (Engel and Grimaldi 2004). By contrast, the number of tarsomeres never exceeds five, but otherwise varies among insect groups. In addition, in some hemimetabolous insects, tarsomere number varies between instars (*e.g.*, Heteroptera), whereas in other groups, different legs may have different numbers of tarsomeres, such as the 5-5-4 pattern of tenebrionoid beetles. In *T. castaneum*, the pretarsus forms a discrete leg segment in the larva, but a single more proximal segment (the tibiotarsus) gives rise to both the tibia and the tarsus of the adult (Figure 1). Thus, tarsal subdivision occurs during appendage metamorphosis. In *D. melanogaster*, the identity of tarsomeres is intercalated between already established pretarsal and tibial identities during mid-to-late third instar imaginal disc development (Kojima *et al.* 2000).

Our data suggest that most genes are conserved in their tarsal patterning roles between *D. melanogaster* and *T. castaneum*, but that several have undergone changes in the PD extent of activity. Genes with highly conserved functions in the tarsus include *dac*, *Dll*, and several genes regulated by *Dll*. Mutations in *dac* in *D. melanogaster* affect tarsomeres 1-3 (t1-t3; Mardon *et al.* 1994), although *dac* is only expressed in t1 (Abu-Shaar and Mann 1998; Lecuit and Cohen 1997), suggesting that the more distal phenotypes result from indirect effects. Similarly, *T. castaneum dac* had a significant bias toward proximal expression in the leg (Figure 2), and *dac* RNAi strongly affected t1-t2 with rarer defects in t3 and t4. The *Dll^Fta^* mutation (Figure 3B) and mildly affected specimens recovered from *Dll* RNAi in *T. castaneum* (Figures 3C and 4C; Suzuki *et al.* 2009) closely resemble weak hypomorphic combinations of *Dll* alleles in *D. melanogaster* (Panganiban 2000). These similar phenotypes suggest that the role of *Dll* as an early activator of tarsus-specific genes is conserved in *T. castaneum*. *Dll* initially activates several genes in a broad tarsal domain, including *bab* and *ss* (reviewed by Kojima 2004). *bab* is required for joint formation within the tarsus in *D. melanogaster* (Godt *et al.* 1993). Similarly, RNAi targeting *bab* during *T. castaneum* metamorphosis caused reduction of the tarsus and loss of joints (Figure 4, T and U). Depletion of *odd*-related genes in *D. melanogaster* and *T. castaneum* also had similar phenotypes in the tarsus. Expression of *ss* is transiently activated in the central tarsus, and *D. melanogaster ss* mutants develop without t2-t4 (Duncan *et al.* 1998; Kozu *et al.* 2006). Knockdown of *ss* in *T. castaneum* produced phenotypes with fusions in the central tarsus, resembling *D. melanogaster* phenotypes (Figure 5).

High levels of EGF signaling at the distal region of the *D. melanogaster* leg disc are also initiated by *Dll* and are required for the development of the pretarsus and distal tarsus; more proximal regions may require a low level of EGF signaling (Campbell 2002; Galindo *et al.* 2002, 2005). *Krn* was also required for development of these structures in *T. castaneum*, where depletion caused deletion of the pretarsus, and reduction and joint loss throughout the tarsus. Frequent defects in the proximal tarsus, deletion of the tibial spurs, and malformation of the distal tibia (Figure 4, R and S) may indicate a more extensive proximal requirement for EGF in the legs of *T. castaneum* compared with *D. melanogaster*. Alternatively, because the *T. castaneum* genome has only one activating EGF ligand (*Krn*), we may detect functions that are redundantly covered by multiple ligands in *D. melanogaster*. In *D. melanogaster*, ultimately, tarsomeres are distinguished by the expression of different combinations of transcription factors, which activate the Notch pathway at each of the intratarsal joints (reviewed by Kojima 2004). The activity of the Notch pathway is also well conserved as discussed above.

EGF activity is key to activating other genes responsible for pretarsus development in *D. melanogaster*, including *Lim1* (Campbell 2005; Galindo *et al.* 2002; Kojima *et al.* 2005; Tsuji *et al.* 2000). Surprisingly, depletion of *Lim1* in *T. castaneum* did not affect distal leg development, although it led to reduction of the femur and tibia and loss of proximal leg joints (Figure 3I), paralleling other aspects of *Lim1* mutant phenotypes in *D. melanogaster* (Pueyo *et al.* 2000). The pretarsus remained unaffected in *T. castaneum* (in 96 of 96 legs scored). One possible explanation for this apparent difference would be that RNAi was performed too late to affect pretarsus development; the claw expression domain is the earliest to appear in *D. melanogaster*, whereas the femur and tibia rings appear last. However, this explanation is insufficient because *Krn* RNAi eliminates the claw and *Krn* is expected to act earlier than *Lim1* if claw development is conserved. Moreover, *Lim1* expression in the leg may be biased proximally (Figure 2; two-tailed *t*-test, *P* = 0.0759).

Another component of the *D. melanogaster* tarsal patterning pathway that appears to have a different domain of effect in *T. castaneum* is *rn*. In *D. melanogaster*, *Dll* activates *rn* in a broad tarsal domain spanning t2-t4 (reviewed by Kojima 2004). Expression of *rn* is transient, but the *rn* null tarsus consists of a single fused segment (Cavener *et al.* 1986; St. Pierre *et al.* 2002). Knockdown of *rn* in *T. castaneum* produced phenotypes that extended more distally than the *D. melanogaster* phenotypes (Figure 5), as loss of the pretarsus was also observed (15 of 95 legs scored). Whether this represents a direct or indirect effect of loss of *rn* expression is unclear.

An interesting aspect of anatomy in *T. castaneum* is the divergence in tarsus segmentation across the legs. *Tribolium* and other tenebrionoid beetles are characterized by having five tarsomeres in the pro- and mesothoracic legs but only four tarsomeres in the metathoracic legs (Figure 1, B−D). This heteromery can be analyzed in the context of our functional genetic data (Figure 5, B and B′). The proximal- and distal-most tarsomeres of the metathoracic tarsus are elongated relative to other tarsomeres. Comparison with serial homologs suggests two alternative hypotheses for how the metathoracic tarsus develops: by the suppression of either the t1/t2 or t4/t5 joint present in the pro- and mesothoracic tarsi. In all three legs, several genes have limits of activity that demarcate the basitarsus (t1). The depletion of *odd*-related paralogs has a high penetrance in t1. Furthermore, *Notch* and *bab* RNAi cause joint loss but not reduction in the proximal tarsomere of all legs. Therefore, it does not appear that the metathoracic tarsus develops by a suppression of the most proximal joint within the tarsus. Serial homology of the distitarsus (t5 in T1-T2; t4 in T3) is supported by the boundaries of function for both *dac* and *dpp*, which are required for the adjacent proximal segments in all legs. These results lead us to reject the hypothesis that the metathoracic tarsus develops by suppression of a presumptive distal joint. The remaining possibility is that the intermediate tarsal region is differently segmented in pro- and mesothoracic *vs.* metathoracic legs. In particular, our data suggest that the presumptive t2/t3 joint is unique to pro- and mesothoracic legs. This hypothesis is supported by the fact that *odd*-related gene knockdown has a relatively high penetrance in the second tarsal joint in pro- and mesothoracic legs but not in metathoracic legs. Conversely, *ab* RNAi causes fusion of tarsal joints with high penetrance, except in the t2/t3 joint of the pro- and mesothoracic legs (*e.g.*, Figure 4V). This hypothesis is also consistent with a model of tarsus segmentation in which intermediate tarsal identities are intercalated between proximal and distal tarsal identities.

### Ontogeny of appendage patterning

The functions of two genes studied here in metamorphic leg patterning have also been examined during embryonic patterning of the larval leg in *T. castaneum*, allowing for comparisons of leg development over ontogeny. The proximal-to-distal extent of *Dll* function is conserved between embryonic and metamorphic patterning. *Dll* mutants (Beermann *et al.* 2001; Figure 3B) and *Dll* RNAi specimens (Suzuki *et al.* 2009; Figure 3, C−E) lack the distal femur and all more distal appendages regions. While *Dll* mutants always have some distal leg identity as adults (Beermann *et al.* 2001), the distal leg can be completely deleted in response to RNAi targeting *Dll* (Figure 3E; Suzuki *et al.* 2009). Presumably, the retention of distal leg identity in *Dll* mutants reflects lethality of stronger *Dll* alleles before the adult stage, whereas RNAi provides a targeted way to reduce its expression at metamorphosis. The deletion of the tibia and tarsus (and the distal femur) in response to *Dll* RNAi also shows that leg segments present in the larval leg can be completely lost at metamorphosis, a result also found in response to *dac* RNAi, in which distal femur, tibia and proximal tarsus are deleted, (Suzuki *et al.* 2009; Figure 3F).

Although *Dll* and *dac* function to pattern and maintain large domains of the leg that persist through all life stages, what about genes involved in uniquely adult features of the leg? Depletion of *ss* during metamorphosis caused fusions or deletions of tarsomeres, but not elsewhere in the leg (Shippy *et al.* 2009; Figure 4Z). In parental *ss* RNAi, larval leg defects were not observed (Shippy *et al.* 2009; Toegel *et al.* 2009). These differences are consistent with the absence of discrete tarsomeres in the larval leg and with their development during metamorphosis.

### The evolution of serially homologous appendages

We have proposed two models representing extreme scenarios for the evolution of developmental mechanisms controlling serially homologous appendages. One model assumes that developmental processes may evolve independently in different serial homologs. However, serial homologs share the same genome making serial homology fundamentally different from special homology (Owen’s terminology, *e.g.*, the leg of *T. castaneum* and the leg of *D. melanogaster*). The evolution of serial homologs may be influenced by the sharing of developmental genes and fitness trade-offs for the organism (Boyden 1947; Wagner 2007), such that serial homologs instead evolve in a constrained, dependent manner.

The independent model predicts that gene functions may differ in the development of only a single appendage type between species. In contrast, the dependent model predicts that changes to the leg-patterning network in a species would also cause changes in the patterning network of other appendages in that same species, leading to greater similarity in patterning of serial homologs within species than special homologs between species. The data presented in this study are considered with results from *D. melanogaster* to test these predictions. Legs are a convenient starting point for such an analysis because their conserved anatomy allows unambiguous determination of whether the PD level of effect has changed across species. For genes with functions that differ in the legs of two species, we then examine whether the gene has a parallel functional difference in any other appendage type. To the extent that serial homologs are developmentally independent within a species, there should be no such parallel changes. To the extent that serial homology stems from shared gene functions, parallel changes are expected.

Several genes function at different PD levels in the legs of *T. castaneum* and *D. melanogaster* (see Figure 5, A and C). *Lim1* RNAi produced defects only in the proximal to intermediate leg in *T. castaneum* (Figure 3I), without the pretarsus defects found in *D. melanogaster Lim1* mutants (Pueyo *et al.* 2000; Tsuji *et al.* 2000). In the adult antennae of *T. castaneum*, *Lim1* was also required for development of the proximal-most segments (Angelini *et al.* 2009), whereas *D. melanogaster Lim1* mutant antennae are either absent, deformed overall, or deformed distally (Pueyo *et al.* 2000). The high sensitivity of distal appendage regions to loss of *Lim1* expression in *D. melanogaster* compared with the insensitivity of these regions to *Lim1* depletion in *T. castaneum* supports the dependent model. A second example comes from *Delta*. In *D. melanogaster* both *Ser* and *Delta* are necessary for joint formation (de Celis *et al.* 1998; Rauskolb and Irvine 1999). However, in *T. castaneum Ser* RNAi caused widespread joint loss, but only 15% of *Delta* RNAi specimens had any joint defects despite 71% reduction in pupal transcript numbers as a result of RNAi (Table 2). Low penetrance for *Delta* RNAi was also seen in the antenna (data not shown) and mouthparts (Angelini *et al.* 2012). Thus, the features of these two genes that are divergent between species are consistent across appendage types. These results underscore the influence of pleiotropy in serial homologs.

However, other genes provide evidence that serial homologs may evolve in a more independent way. *hth* functions as a regulator of proximal identity in both *T. castaneum* and *D. melanogaster* legs, but the extent of proximal reduction differs between these species. When lacking *hth* activity, the leg discs of *D. melanogaster* develop a normal tarsus, but they have a single fused proximal segment (Casares and Mann 2001). Thus, *hth* is required for cell growth and maintenance in the proximal leg of *D. melanogaster*. A similar role for *hth* in leg development is known for a more distantly related species, the cricket *Gryllus bimaculatus* (Ronco *et al.* 2008). By contrast, all of the primary leg segments are retained in response to *hth* depletion in *T. castaneum*, although the identity of the coxa, trochanter and femur is affected (Figure 3J). However in other *T. castaneum* appendage types, *hth* has a role in both growth and patterning. In the maxillae and labium *hth* knockdown causes proximal transformations, but in severely affected individuals a proximal segment of the palps was deleted (Angelini *et al.* 2012). Moreover, *hth* RNAi produced strong reductions in antenna length, through fusions or loss of the intermediate segments (Angelini *et al.* 2009). In addition, RNAi targeting *rn* in *T. castaneum* produced defects in the pretarsus, which is not seen in *D. melanogaster rn* mutants (Cavener *et al.* 1986; St. Pierre *et al.* 2002). However *rn* loss-of-function in the antenna results in defects in the intermediate funicle region in *T. castaneum* (Angelini *et al.* 2009) and the intermediate (a3-a4) segments in *D. melanogaster* (Cavener *et al.* 1986). No defects are seen in the distal-most antenna structures of either species. Therefore, the novel aspects of *hth* and *rn* function are unique to specific appendage types.

In the case of EGF signaling, differences were observed across species, but in a pattern that is potentially consistent with either hypothesis, depending on the ancestral state. *Krn* was required in a broader domain including more proximal structures in the legs of *T. castaneum* compared to the relatively distal requirement for EGF signaling in *D. melanogaster* legs (Campbell 2002). *Krn* was also required throughout the antenna (Angelini *et al.* 2009), and maxillary and labial palps of *T. castaneum* (Angelini *et al.* 2012) at metamorphosis. In contrast, in *D. melanogaster*, loss of EGF receptor function has not produced described defects in antennal PD patterning (Amin and Finkelstein 1999; Clifford and Schupbach 1989).

These functional comparisons among serial homologs reveal some support for each hypothesis. Although negative results, such as the absence of phenotypes in the pretarsus for *Lim1* RNAi, low penetrance for *Delta* knockdown, and the lack of leg reduction in *hth* RNAi, are relatively weak support for each hypothesis, positive results, such as the novel function for *rn* in *T. castaneum*, provide stronger support. Complete dependence or independence in the evolution of serial homologs is not expected. Instead our data suggest a complex mix of divergence and constraint among appendages. Despite the breadth of this study, we still have only a limited number of comparisons. Data from other genes and taxa also suggest mixed support for the two hypotheses. For example, *pdm*/*nubbbin* has exceptionally labile expression (Li and Popadić 2004) and functional domains (Hrycaj *et al.* 2008; Turchyn *et al.* 2011) among insects, but there is some consistency across special homologs, as predicted by the independent model. More extensive comparative functional studies will be necessary in the future to evaluate the extent of developmental dependence among serial homologs, and to examine whether genes involved in certain developmental processes are more prone to divergence in limited or universal ways.

### Conclusion

Serial homology has been considered in biology for more than two centuries, and it is characterized by the influence of shared developmental processes or pleiotropy (Boyden 1947; Owen 1848; Roth 1984; Wagner 2007). On one hand, shared development indicates that serial homology presents an accessible pathway through which novel structures may originate. On the other hand, shared development may constrain the subsequent evolution of serial homologs. To the extent that selection favors divergence in the function of serial homologs, any genes deployed in these structures may be subject to antagonistic pleiotropy. Developmental changes favored by selection on a structure may result in developmental changes of the serially homologous structures at other positions in the body. Thus, it is likely that trade-offs resulting from pleiotropy bias the mutations that are ultimately fixed by selection during evolution (Stern and Orgogozo 2009). The prevalence of this influence remains an important gap in our knowledge of evolution. This study has made a small-scale test of the question here, finding that changes in leg patterning between groups are often accompanied by parallel changes in other appendages. Comparative studies in diverse species and other types of serially homologous structures will help to resolve the issue.

## Supplementary Material

Supporting Information

## References

[bib1] Abu-Shaar M. Mann R. S., 1998 Generation of multiple antagonistic domains along the proximodistal axis during *Drosophila* leg development. Development 125: 3821–3830 9729490 10.1242/dev.125.19.3821

[bib2] Abzhanov A. Kaufman T. C., 2000 Homologs of *Drosophila* appendage genes in the patterning of arthropod limbs. Dev. Biol. 227: 673–689 11071783 10.1006/dbio.2000.9904

[bib3] Amin A. Li Y. Finkelstein R., 1999 Hedgehog activates the EGF receptor pathway during *Drosophila* head development. Development 126: 2623–2630 10331974 10.1242/dev.126.12.2623

[bib4] Angelini D. R. Kaufman T. C., 2004 Functional analyses in the hemipteran *Oncopeltus fasciatus* reveal conserved and derived aspects of appendage patterning in insects. Dev. Biol. 271: 306–321 15223336 10.1016/j.ydbio.2004.04.005

[bib5] Angelini D. R. Kaufman T. C., 2005 a Functional analyses in the milkweed bug *Oncopeltus fasciatus* (Hemiptera) support a role for Wnt signaling in body segmentation but not appendage development. Dev. Biol. 283: 409–423 15939417 10.1016/j.ydbio.2005.04.034

[bib6] Angelini D. R. Kaufman T. C., 2005 b Insect appendages and comparative ontogenetics. Dev. Biol. 286: 57–77 16112665 10.1016/j.ydbio.2005.07.006

[bib7] Angelini D. R. Kikuchi M. Jockusch E. L., 2009 Genetic patterning in the adult capitate antenna of the beetle *Tribolium castaneum*. Dev. Biol. 327: 240–251 19059230 10.1016/j.ydbio.2008.10.047

[bib8] Angelini D. R. Smith F. W. Aspiras A. C. Kikuchi M. Jockusch E. L., 2012 Patterning of adult mandibulate mouthparts in the red flour beetle, *Tribolium castaneum*. Genetics 190: 639–654 10.1534/genetics.111.134296 PMC3276642 22135350

[bib86] Aspiras A. C. Smith F. W. Angelini D. R., 2011 Sex-specific gene interactions in the patterning of insect genitalia. Dev. Biol. 360: 369–380 21996282 10.1016/j.ydbio.2011.09.026

[bib9] Beermann A. Jay D. G. Beeman R. W. Hulskamp M. Tautz D., 2001 The *Short antennae* gene of *Tribolium* is required for limb development and encodes the orthologue of the *Drosophila* Distal-less protein. Development 128: 287–297 11124123 10.1242/dev.128.2.287

[bib10] Bishop S. A. Klein T. Arias A. M. Couso J. P., 1999 Composite signalling from *Serrate* and *Delta* establishes leg segments in *Drosophila* through *Notch*. Development 126: 2993–3003 10357942 10.1242/dev.126.13.2993

[bib11] Boyden A., 1947 Homology and analogy. A critical review of the meanings and implications of these concepts in biology. Am. Midl. Nat. 37: 648–669

[bib12] Boxshall G. A., 2004 The evolution of arthropod limbs. Biol. Rev. Camb. Philos. Soc. 79: 253–300 15191225 10.1017/s1464793103006274

[bib13] Campbell G., 2002 Distalization of the *Drosophila* leg by graded EGF-receptor activity. Nature 418: 781–785 12181568 10.1038/nature00971

[bib14] Campbell G., 2005 Regulation of gene expression in the distal region of the *Drosophila* leg by the *Hox11* homolog, *C15*. Dev. Biol. 278: 607–618 15680373 10.1016/j.ydbio.2004.12.009

[bib15] Casares F. Mann R. S., 2001 The ground state of the ventral appendage in *Drosophila*. Science 293: 1477–1480 11520984 10.1126/science.1062542

[bib16] Cavener D. R. Otteson D. C. Kaufman T. C., 1986 A rehabilitation of the genetic map of the 84B-D region in *Drosophila melanogaster*. Genetics 114: 111–123 3095179 10.1093/genetics/114.1.111 PMC1202924

[bib17] Chu J. Dong P. D. S. Panganiban G., 2002 Limb type-specific regulation of *bric a brac* contributes to morphological diversity. Development 129: 695–704 11830570 10.1242/dev.129.3.695

[bib18] Clifford R. J. Schupbach T., 1989 Coordinately and differentially mutable activities of *torpedo*, the *Drosophila melanogaster* homolog of the vertebrate EGF receptor gene. Genetics 123: 771–787 2515109 10.1093/genetics/123.4.771 PMC1203888

[bib19] Cohen S. M. Jürgens G., 1989 Proximal-distal pattern formation in *Drosophila*: graded requirement for *Distal-less* gene activity during limb development. Roux’s Arch. Dev. Biol. 198: 157–169 10.1007/BF02438941 28305718

[bib20] Couderc J.-L. Godt D. Zollman S. Chen J. Li M., 2002 The *bric à brac* locus consists of two paralogous genes encoding BTB/POZ domain proteins and acts as a homeotic and morphogenetic regulator of imaginal development in *Drosophila*. Development 129: 2419–2433 11973274 10.1242/dev.129.10.2419

[bib21] Daly H. V. Doyen J. T. Purcell A. H. I., 1998 Introduction to Insect Biology and Diversity. Oxford University Press, Oxford

[bib22] Darwin C., 1859 On The Origin of Species by Means of Natural Selection. Murray, London

[bib23] De Celis Ibeas J. M. Bray S. J., 2003 Bowl is required downstream of Notch for elaboration of distal limb patterning. Development 130: 5943–5952 14573519 10.1242/dev.00833

[bib24] De Celis J. F. Tyler D. M. De Celis J. Bray S. J., 1998 Notch signalling mediates segmentation of the *Drosophila* leg. Development 125: 4617–4626 9806911 10.1242/dev.125.23.4617

[bib25] Dehal P. Boore J. L., 2005 Two rounds of whole genome duplication in the ancestral vertebrate. PLoS Biol. 3: e314 16128622 10.1371/journal.pbio.0030314 PMC1197285

[bib26] Diaz-Benjumea F. J. Cohen B. Cohen S. M., 1994 Cell interaction between compartments establishes the proximal-distal axis of *Drosophila* legs. Nature 372: 175–179 7969450 10.1038/372175a0

[bib27] Duncan D. M. Burgess E. A. Duncan I., 1998 Control of distal antennal identity and tarsal development in *Drosophila* by *spineless*-*aristapedia*, a homolog of the mammalian dioxin receptor. Genes Dev. 12: 1290–1303 9573046 10.1101/gad.12.9.1290 PMC316766

[bib28] Engel M. S. Grimaldi D. A., 2004 New light shed on the oldest insect. Nature 427: 627–630 14961119 10.1038/nature02291

[bib29] Galindo M. I. Bishop S. A. Greig S. Couso J. P., 2002 Leg patterning driven by proximal-distal interactions and EGFR signaling. Science 297: 256–259 12114628 10.1126/science.1072311

[bib30] Galindo M. I. Bishop S. A. Couso J. P., 2005 Dynamic EGFR-Ras signalling in *Drosophila* leg development. Dev. Dyn. 233: 1496–1508 15965980 10.1002/dvdy.20452

[bib31] Goethe J. W. v., 1790 *Versuch die Metamorphose der Pflanzen zu erklaren.* Ettingersche Buchhandlung, Gotha

[bib32] Godt D. Couderc J. L. Cramton S. E. Laski F. A., 1993 Pattern formation in the limbs of *Drosophila*: *bric a brac* is expressed in both a gradient and a wave-like pattern and is required for specification and proper segmentation of the tarsus. Development 119: 799–812 7910551 10.1242/dev.119.3.799

[bib33] Greenberg L. Hatini V., 2009 Essential roles for *lines* in mediating leg and antennal proximodistal patterning and generating a stable Notch signaling interface at segment borders. Dev. Biol. 330: 93–104 19324031 10.1016/j.ydbio.2009.03.014 PMC2917052

[bib34] Hao I. Green R. B. Dunaevsky O. Lengyel J. A. Rauskolb C., 2003 The *odd-skipped* family of zinc finger genes promotes *Drosophila* leg segmentation. Dev. Biol. 263: 282–295 14597202 10.1016/j.ydbio.2003.07.011

[bib35] Hrycaj S. Mihajlovic M. Mahfooz N. Couso J. P. Popadić A., 2008 RNAi analysis of *nubbin* embryonic functions in a hemimetabolous insect, *Oncopeltus fasciatus*. Evol. Dev. 10: 705–716 19021741 10.1111/j.1525-142X.2008.00284.x

[bib36] Hu S. Fambrough D. Atashi J. R. Goodman C. S. Crews S. T., 1995 The *Drosophila abrupt* gene encodes a BTB-zinc finger regulatory protein that controls the specificity of neuromuscular connections. Genes Dev. 9: 2936–2948 7498790 10.1101/gad.9.23.2936

[bib37] Huet C. Lenoir-Rousseaux J. J., 1976 Etude de la mise en place de la patte imaginable de *Tenebrio molitor*. 1. Analyse expérimentale des processus de restauration au cours de la morphogenése. J. Embryol. Exp. Morphol. 35: 303–321 939941

[bib38] Inoue Y. Mito T. Miyawaki K. Matsushima K. Shinmyo Y., 2002 Correlation of expression patterns of *homothorax*, *dachshund*, and *Distal-less* with the proximodistal segmentation of the cricket leg bud. Mech. Dev. 113: 141–148 11960702 10.1016/s0925-4773(02)00017-5

[bib39] Jockusch E. L. Nulsen C. Newfeld S. J. Nagy L. M., 2000 Leg development in flies *vs.* grasshoppers: differences in *dpp* expression do not lead to differences in the expression of downstream components of the leg patterning pathway. Development 127: 1617–1626 10725238 10.1242/dev.127.8.1617

[bib40] Jockusch E. L. Williams T. A. Nagy L. M., 2004 The evolution of patterning of serially homologous appendages in insects. Dev. Genes Evol. 214: 324–338 15170569 10.1007/s00427-004-0412-6

[bib41] Keilin D., 1915 Recherches sur les larves de diptères cyclorhaphes. Bull. Sci. Fr. Belg. 49: 15–198

[bib42] Kojima T., 2004 The mechanism of *Drosophila* leg development along the proximodistal axis. Dev. Growth Differ. 46: 115–129 15066191 10.1111/j.1440-169X.2004.00735.x

[bib43] Kojima T. Sato M. Saigo K., 2000 Formation and specification of distal leg segments in *Drosophila* by dual *Bar* homeobox genes, *BarH1* and *BarH2*. Development 127: 769–778 10648235 10.1242/dev.127.4.769

[bib44] Kojima T. Tsuji T. Saigo K., 2005 A concerted action of a paired-type homeobox gene, *aristaless*, and a homolog of *Hox11/tlx* homeobox gene, *clawless*, is essential for the distal tip development of the *Drosophila* leg. Dev. Biol. 279: 434–445 15733670 10.1016/j.ydbio.2004.12.005

[bib45] Kozu S. Tajiri R. Tsuji T. Michiue T. Saigo K., 2006 Temporal regulation of late expression of *Bar* homeobox genes during *Drosophila* leg development by Spineless, a homolog of the mammalian dioxin receptor. Dev. Biol. 294: 497–508 16631729 10.1016/j.ydbio.2006.03.015

[bib46] Lecuit T. Cohen S. M., 1997 Proximal-distal axis formation in the *Drosophila* leg. Nature 388: 139–145 9217152 10.1038/40563

[bib47] Li H. Popadić A., 2004 Analysis of *nubbin* expression patterns in insects. Evol. Dev. 6: 310–324 15330864 10.1111/j.1525-142X.2004.04039.x

[bib48] Lilly B. O’Keefe D. D. Thomas J. B. Botas J., 1999 The LIM homeodomain protein dLim1 defines a subclass of neurons within the embryonic ventral nerve cord of *Drosophila*. Mech. Dev. 88: 195–205 10534618 10.1016/s0925-4773(99)00189-6

[bib49] Lynch M. Force A., 2000 The probability of duplicate gene preservation by subfunctionalization. Genetics 154: 459–473 10629003 10.1093/genetics/154.1.459 PMC1460895

[bib50] Mardon G. Solomon N. M. Rubin G. M., 1994 *dachshund* encodes a nuclear protein required for normal eye and leg development in *Drosophila*. Development 120: 3473–3486 7821215 10.1242/dev.120.12.3473

[bib51] Moczek A. P. Rose D. J., 2009 Differential recruitment of limb patterning genes during development and diversification of beetle horns. Proc. Natl. Acad. Sci. USA 106: 8992–8997 19451631 10.1073/pnas.0809668106 PMC2690047

[bib52] Niimi T. Kuwayama H. Yaginuma T., 2005 Larval RNAi applied to the analysis of postembryonic development in the ladybird beetle, *Harmonia axyridis*. J. Insect Biotechnol. Sericology 74: 95–102

[bib53] Ober K. A. Jockusch E. L., 2006 The roles of *wingless* and *decapentaplegic* in axis and appendage development in the red flour beetle, *Tribolium castaneum*. Dev. Biol. 294: 391–405 16616738 10.1016/j.ydbio.2006.02.053

[bib54] Ohno S., 1970 Evolution by Gene Duplication. Springer-Verlag, Berlin

[bib55] Owen R., 1848 On the Archetype and Homologies of the Vertebrate Skeleton. Richard and John E. Taylor, London

[bib56] Palopoli M. F. Patel N. H., 1998 Evolution of the interaction between Hox genes and a downstream target. Curr. Biol. 8: 587–590 9601643 10.1016/s0960-9822(98)70228-3

[bib57] Panganiban G., 2000 *Distal-less* function during *Drosophila* appendage and sense organ development. Dev. Dyn. 218: 554–562 10906775 10.1002/1097-0177(200008)218:4<554::AID-DVDY1023>3.0.CO;2-#

[bib58] Panganiban G. Nagy L. Carroll S. B., 1994 The role of the *Distal-less* gene in the development and evolution of insect limbs. Curr. Biol. 4: 671–675 7953552 10.1016/s0960-9822(00)00151-2

[bib59] Pfaffl M. W., 2001 A new mathematical model for relative quantification in real-time RT-PCR. Nucleic Acids Res. 29: e45 11328886 10.1093/nar/29.9.e45 PMC55695

[bib60] Prpic N.-M. Damen W. G. M., 2009 *Notch*-mediated segmentation of the appendages is a molecular phylotypic trait of the arthropods. Dev. Biol. 326: 262–271 19046962 10.1016/j.ydbio.2008.10.049

[bib87] Prpic N. M. Wigand B. Damen W. G. Klingler M., 2011 Expression of *dachshund* in wild-type and *Distal-less* mutant *Tribolium* corroborates serial homologies in insect appendages. Dev. Genes Evol. 211: 467–477 11702196 10.1007/s004270100178

[bib61] Pueyo J. I. Galindo M. I. Bishop S. A. Couso J. P., 2000 Proximal-distal leg development in *Drosophila* requires the *apterous* gene and the *Lim1* homologue *dlim1*. Development 127: 5391–5402 11076760 10.1242/dev.127.24.5391

[bib62] Rauskolb C., 2001 The establishment of segmentation in the *Drosophila* leg. Development 128: 4511–4521 11714676 10.1242/dev.128.22.4511

[bib63] Rauskolb C. Irvine K. D., 1999 Notch-mediated segmentation and growth control of the *Drosophila* leg. Dev. Biol. 210: 339–350 10357895 10.1006/dbio.1999.9273

[bib88] Rogers B. T. Peterson M. D. Kaufman T. C., 2002 The development and evolution of insect mouthparts as revealed by the expression Patterns of Gnathocephalic genes. Evol. Dev. 4: 96–110 12004967 10.1046/j.1525-142x.2002.01065.x

[bib64] Roth V. L., 1984 On homology. Biol. J. Linn. Soc. Lond. 22: 13–29

[bib65] Ronco M. Uda T. Mito T. Minelli A. Noji S., 2008 Antenna and all gnathal appendages are similarly transformed by *homothorax* knock-down in the cricket *Gryllus bimaculatus*. Dev. Biol. 313: 80–92 18061158 10.1016/j.ydbio.2007.09.059

[bib89] Sanchez-Salazar J. Pletcher M. T. Bennett R. L. Brown S. J. Dandamudi T. J., 1996 The *Tribolium decapentaplegic* gene is similar in sequence, structure, and expression to the *Drosophila dpp* gene. Dev. Genes Evol. 206: 237–246 24173563 10.1007/s004270050049

[bib66] Schoppmeier M. Damen W. G., 2001 Double-stranded RNA interference in the spider *Cupiennius salei*: the role of *Distal-less* is evolutionarily conserved in arthropod appendage formation. Dev. Genes Evol. 211: 76–82 11455417 10.1007/s004270000121

[bib67] Shippy T. Yeager S. Denell R., 2009 The *Tribolium spineless* ortholog specifies both larval and adult antennal identity. Dev. Genes Evol. 219: 45–51 19030877 10.1007/s00427-008-0261-9 PMC2605184

[bib68] Snodgrass R. E., 1935 Principles of Insect Morphology. McGraw-Hill, New York

[bib69] Sokoloff A., 1972 The Biology of Tribolium, Vol. 1 Clarendon Press, Oxford

[bib70] Stern D. L. Orgogozo V., 2009 Is genetic evolution predictable? Science 323: 746–751 19197055 10.1126/science.1158997 PMC3184636

[bib71] St. Pierre S. E. Galindo M. I. Couso J. P. Thor S., 2002 Control of *Drosophila* imaginal disc development by *rotund* and *roughened eye*: differentially expressed transcripts of the same gene encoding functionally distinct zinc finger proteins. Development 129: 1273–1281 11874922 10.1242/dev.129.5.1273

[bib72] Suzuki Y. Squires D. C. Riddiford L. M., 2009 Larval leg integrity is maintained by *Distal-less* and is required for proper timing of metamorphosis in the flour beetle, *Tribolium castaneum*. Dev. Biol. 326: 60–67 19022238 10.1016/j.ydbio.2008.10.022 PMC2762819

[bib73] Svacha P., 1992 What are and what are not imaginal discs: reevaluation of some basic concepts (Insecta, Holometabola). Dev. Biol. 154: 101–117 1426619 10.1016/0012-1606(92)90052-i

[bib74] Tanaka K. Truman J. W., 2005 Development of the adult leg epidermis in *Manduca sexta*: contribution of different larval cell populations. Dev. Genes Evol. 215: 78–89 15647943 10.1007/s00427-004-0458-5

[bib75] Theisen H. Haerry T. E. O'connor M. B. Marsh J. L., 1996 Developmental territories created by mutual antagonism between Wingless and Decapentaplegic. Development 122: 3939–3948 9012514 10.1242/dev.122.12.3939

[bib76] Toegel J. Wimmer E. Prpic N.-M., 2009 Loss of *spineless* function transforms the *Tribolium* antenna into a thoracic leg with pretarsal, tibiotarsal, and femoral identity. Dev. Genes Evol. 219: 53–58 19030876 10.1007/s00427-008-0265-5

[bib77] Tomoyasu Y. Denell R. E., 2004 Larval RNAi in *Tribolium* (Coleoptera) for analyzing adult development. Dev. Genes Evol. 214: 575–578 15365833 10.1007/s00427-004-0434-0

[bib78] Tribolium Genome Sequencing Consortium, 2008 The genome of the model beetle and pest *Tribolium castaneum*. Nature 452: 949–955 18362917 10.1038/nature06784

[bib79] True J. R. Haag E. S., 2001 Developmental system drift and flexibility in evolutionary trajectories. Evol. Dev. 3: 109–119 11341673 10.1046/j.1525-142x.2001.003002109.x

[bib80] Tsuji T. Sato A. Hiratani I. Taira M. Saigo K., 2000 Requirements of *Lim1*, a *Drosophila* LIM-homeobox gene, for normal leg and antennal development. Development 127: 4315–4323 11003832 10.1242/dev.127.20.4315

[bib90] Turchyn N. Chesebro J. Hrycaj S. Couso J. P. Popadić A., 2011 Evolution of *nubbin* function in hemimetabolous and holometabolous insect appendages. Dev. Biol. 357: 83–95 21708143 10.1016/j.ydbio.2011.06.014 PMC3178182

[bib81] van der Meer J. M., 1977 Optical clean and permanent whole mount preparation for phase-contrast microscopy of cuticular structures of insect larvae. Dros. Inf. Serv. 52: 160

[bib82] van der Zee M. Stockhammer O. Von Levetzow C. Nunes Da Fonseca R. Roth S., 2006 Sog/Chordin is required for ventral-to-dorsal Dpp/BMP transport and head formation in a short germ insect. Proc. Natl. Acad. Sci. USA 103: 16307–16312 17050690 10.1073/pnas.0605154103 PMC1637578

[bib83] Wagner G. P., 2007 The developmental genetics of homology. Nat. Rev. Genet. 8: 473–479 17486120 10.1038/nrg2099

[bib84] Wu J. Cohen S. M., 1999 Proximodistal axis formation in the *Drosophila* leg: subdivision into proximal and distal domains by *homothorax* and *Distal-less*. Development 126: 109–117 9834190 10.1242/dev.126.1.109

[bib85] Zeng C. Justice N. J. Abdelilah S. Chan Y. M. Jan L. Y., 1998 The Drosophila LIM-only gene, dLMO, is mutated in Beadex alleles and might represent an evolutionarily conserved function in appendage development. Proc. Natl. Acad. Sci. USA 95: 10637–10642 9724756 10.1073/pnas.95.18.10637 PMC27947

