# Supplementary material for: Extent With Modification: Leg Patterning in the Beetle Tribolium castaneum and the Evolution of Serial Homologs
Source: G3 (Bethesda). 2012 Feb 1;2(2):235–48. doi: 10.1534/g3.111.001537 (PMC3284331; doi:10.1534/g3.111.001537)
Supplement: Supporting Information [file supp_2_2_235__index.html]

Supporting Information 

# Extent With Modification: Leg Patterning in the Beetle *Tribolium castaneum* and the Evolution of Serial Homologs

## Supporting Information for Angelini *et al.*, 2012

**Files in this Data Supplement:**

- File S1 - Supporting data (.xls, 561 KB)
